# Non-HIV *Pneumocystis* pneumonia: do conventional community-acquired pneumonia guidelines under estimate its severity?

**DOI:** 10.1186/2049-6958-7-2

**Published:** 2012-06-11

**Authors:** Nobuhiro Asai, Shinji Motojima, Yoshihiro Ohkuni, Ryo Matsunuma, Kei Nakasima, Takuya Iwasaki, Tamao Nakashita, Yoshihito Otsuka, Norihiro Kaneko

**Affiliations:** 1 Department of Pulmonology, Kameda Medical Center, 296-8602, 929 higashi-cho, Kamogawa-city, Chiba, Japan; 2 Department of Rheumatology, Kameda Medical Center, 296-8602, 929 higashi-cho, Kamogawa-city, Chiba, Japan; 3 Laboratory medicine, Kameda Medical Center, 296-8602, 929 higashi-cho, Kamogawa-city, Chiba, Japan

**Keywords:** Community acquired pneumonia, Guidelines, Mortality, Non-HIV *Pneumocystis* pneumonia

## Abstract

**Background:**

Non-HIV *Pneumocystis* pneumonia (PCP) can occur in immunosuppressed patients having malignancy or on immunosuppressive agents. To classify severity, the A-DROP scale proposed by the Japanese Respiratory Society (JRS), the CURB-65 score of the British Respiratory Society (BTS) and the Pneumonia Severity Index (PSI) of the Infectious Diseases Society of America (IDSA) are widely used in patients with community-acquired pneumonia (CAP) in Japan. To evaluate how correctly these conventional prognostic guidelines for CAP reflect the severity of non-HIV PCP, we retrospectively analyzed 21 patients with non-HIV PCP.

**Methods:**

A total of 21 patients were diagnosed by conventional staining and polymerase chain reaction (PCR) for respiratory samples with chest x-ray and computed tomography (CT) findings. We compared the severity of 21 patients with PCP classified by A-DROP, CURB-65, and PSI. Also, patients’ characteristics, clinical pictures, laboratory results at first visit or admission and intervals from diagnosis to start of specific-PCP therapy were evaluated in both survivor and non-survivor groups.

**Results:**

Based on A-DROP, 18 patients were classified as mild or moderate; respiratory failure developed in 15 of these 18 (83.3%), and 7/15 (46.7%) died. Based on CURB-65, 19 patients were classified as mild or moderate; respiratory failure developed in 16/19 (84.2%), and 8 of the 16 (50%) died. In contrast, PSI classified 14 as severe or extremely severe; all of the 14 (100%) developed respiratory failure and 8/14 (57.1%) died. There were no significant differences in laboratory results in these groups. The time between the initial visit and diagnosis, and the time between the initial visit and starting of specific-PCP therapy were statistically shorter in the survivor group than in the non-survivor group.

**Conclusions:**

Conventional prognostic guidelines for CAP could underestimate the severity of non-HIV PCP, resulting in a therapeutic delay resulting in high mortality. The most important factor to improve the mortality of non-HIV PCP is early diagnosis and starting of specific-PCP therapy as soon as possible.

## Background

*Pneumocystis* pneumonia (PCP) not related to human immunodeficiency virus (HIV) can occur in immunosuppressed patients having malignancy or on immunosuppressive agents [1-10]. The mortality of patients with PCP without HIV infections ranges from 0 to 70% [1,3-10], compared to that of HIV-infected PCP patients, which ranges from 10 to 20% [1,4,9]. Besides, mortality rates as high as 60–75% have been reported in PCP patients without AIDS who required mechanical ventilation [11,12]. The higher mortality among non-HIV PCP patients has been attributed to severe lung inflammation [1,4,10], although the exact etiology accounting for these large differences in mortality has not yet been determined.

The A-DROP system proposed by the Japanese Respiratory Society (JRS), the CURB-65 score proposed by British Respiratory Society (BTS) and the Pneumonia Severity Index (PSI) proposed by the Infectious of Disease Society of America (IDSA) are widely used in classifying patients with community-acquired pneumonia (CAP) [13-15] in Japan. For the purpose of evaluating how correctly the conventional prognostic guidelines of CAP reflect the severity of non-HIV PCP, we retrospectively analyzed 21 patients with non-HIV PCP. It has never previously been reported that the severity of non-HIV PCP may be underestimated by these prognostic guidelines. This is the first report focusing on the limit of conventional prognostic guidelines of CAP for non-HIV PCP.

## Methods

From the end of 2009 to the beginning of 2010 we retrospectively reviewed all the cases of PCP diagnosed as CAP in the Kameda Medical Center, Chiba, Japan. All the patients had undergone HIV testing and were negative. Patients with hospital associated pneumonia (HAP) were excluded. PCP was diagnosed based on polymerase chain reaction (PCR) and conventional PCP staining with Grocott methenamine silver stain or Diff-Quick™ staining in respiratory samples such as induced sputum (IS) or bronchoalveolar lavage (BAL) fluid associated with radiographic infiltration on chest X-ray and computed tomography (CT) findings on admission. All radiographic pictures showed infiltration confirmed by the pulmonologist and radiologist in our hospital. The decision to perform IS or BAL examination depended on the patient’s general condition at the discretion of attending physicians. PCP diagnosis was based not solely on the positive PCR respiratory specimen but also on the clinical and radiological findings consistent with the diagnosis of PCP as well as complete recovery with anti-*Pneumocystis jirovecii* treatment alone. No biopsy was performed during this study. We compared the severity of 21 patients with PCP classified by A-DROP, CURB-65, and PSI, and analyzed the background and laboratory data of each.

### Statistical analysis

Comparisons of group means were made by unpaired or paired *t*-tests or Mann–Whitney *U*-test. Contingency tables were evaluated by Fisher’s exact probability test. *p* values < 0.05 were considered significant.

## Results

### Patient characteristics

The characteristics of the 21 patients with PCP are shown in Tables 1 and 2. The mean age was 71.5 years (range 57–88). Twelve patients (57.1%) had rheumatic or autoimmune disease which was the most common underlying disease, followed by malignancy (n = 10, 47.6%). Seventeen patients (77.8%) were receiving steroid or immunosuppressants for the underlying disease. Prophylactic therapy consisting of trimethoprim-sulfamethoxazole (TMP/SMX) was used by only 1 patient (4.8%).

**Table 1:** Characteristics of PCP patients without HIV infection (n = 21)

Characteristics	Patients	
	n	%
Sex		
Male	11	52.4
Female	10	47.6
Underlying disease		
Rheumatic and autoimmune diseases	12	57.1
Malignancy	10	47.6
Diabetes mellitus	4	19.0
Chronic pulmonary diseases	6	28.6
Heart diseases	4	19.0
Cerebrovascular diseases	2	9.5
Renal diseases	2	9.5
Liver disease	2	9.5
Long-term glucocorticoids alone	4	19.0
Immunosuppressants alone /chemotherapeutic agents alone	6	28.6
Long-term glucocorticoids combined with chemotherapeutic/immunosuppressive agents	7	33.3
PCP prophylaxis	1	4.8

Table 1 legend - PCP, *Pneumocystis* pneumonia; SD, standard deviation.

**Table 2:** Relationship between complications and outcome

Complications	Survivors (n = 13)	Non-survivors (n = 8)	p
Malignancy	4	6	0.081
Rheumatic and autoimmune disease	9	3	0.203
Diabetes Mellitus	2	1	1.000
Chronic pulmonary disease	3	3	0.631
Heart disease	2	2	0.618
Cerebrovascular disease	0	2	0.133
Renal disease	1	1	1.000
Liver disease	2	0	0.505

Table 2 legend -Presented data are evaluated by Fisher’s exact test.

### Treatment and outcomes

All the patients received oral or parenteral (TMP/SMX and adjuvant steroid therapy. Eighteen (85.7%) of the 21 patients had acute respiratory failure. Eight patients (38.1%) in the study died.

### Severity of PCP by A-DROP

The A-DROP scale was one of the prognostic guidelines for CAP proposed by JRS in 2005. It is a scoring system using age, degree of dehydration (serum blood urea nitrogen (BUN)), SpO_2_ < 90%(PaO_2_ < 60 mm Hg), orientation, and systolic blood pressure. All our patients were classified by the A-DROP system. As a result, 18 patients (85.7%) were classified as mild or moderate. Fifteen (83.3%) of the 18 developed respiratory failure, and 7 (46.7%) of the 15 died (Table 3).

**Table 3:** Distribution of patients and their mortality according to risk class

Risk groups	Number of patients (%)	Number of patients with respiratory failure (%)	Mortality (%)
A-DROP 0	5 (23.8)	3 (60)	0 (0)
1	6 (28.6)	5 (83.3)	2 (33.3)
2	7 (33.3)	7 (100)	5 (71.4)
3	2 (9.5)	2 (100)	1 (50)
4/5	1 (4.8)	1 (100)	0 (0)
CURB-65 0	4 (19)	2 (50)	0 (0)
1	3 (14.3)	2 (66.7)	0 (0)
2	12 (57.1)	12 (100)	8 (66.7)
3	0 (0)		
4/5	2 (9.5)	2 (100)	0 (0)
PSI^*^ I	0 (0)		
II	4 (19)	2 (50)	0 (0)
III	3 (14.3)	2 (66.7)	1 (33.3)
IV	12 (57.1)	12 (100)	6 (50)
V	2 (9.5)	2 (100)	1 (50)

*In PSI, relations of risk classes to net points are as follows:

I: 0, II: 1–70, III: 71–90, IV: 91–130, V: >131.

Table 3 legend - PCP, *Pneumocystis* pneumonia; PSI, pulmonary severity index.

### Severity of PCP by CURB-65

CURB-65 is also a prognostic guideline for CAP, proposed by BTS in 1996. It seems to be similar to the A-DROP system, the greatest difference being the age value used in the scoring. While JRS defines males > 70 years and females > 75 years as high risk elderly, BTS classifies high risk elderly age as > 65 years for both sexes. In our study 19 patients (90.5%) were classified as mild or moderate (class 0–2); 16 of the 19 patients (84.2%) developed acute respiratory failure, and 8 (42.1%) of the 16 died (Table 3).

### Severity of PCP by PSI

The PSI was proposed by the Infectious Diseases Society of America/American Thoracic Society (IDSA/ATS). It is considered to be more complicated and less convenient to use than the other scoring systems. It consists of 19 items such as age, underlying disease, gender, vital signs, etc. In our study, 14 patients (66.7%) were classified as severe or extremely severe by PSI. All the 14 patients developed respiratory failure and 7 patients (50%) died (Table 3).

### Distribution of patients and their mortality in each risk class of A-DROP, CURB-65 and PSI

The majority of the PCP patients were categorized in risk classes 2–3 by A-DROP and CURB-65, and III/IV by PSI. Of note are the very high patient mortalities in risk classes 2 by A-DROP and CURB-65, and class III/IV by PSI (Table 3).

### Comparison of the prognostic accuracy between A-DROP, CURB-65 and PSI

The comparison of the prognostic accuracy of each guideline for CAP is shown in Table 4. It can be seen that the positive predictive values and negative predictive values for mortality in each system were low.

**Table 4:** Comparison of the prognostic accuracy of the A-DROP system and CURB-65 score and PSI

Sensitivity for mortality, %			
	A-DROP scores	3–5	12.5
	CURB-65 scores	3–5	0
	PSI risk classes	IV- V	87.5
Specificity for mortality, %			
	A-DROP scores	3–5	84.6
	CURB-65 scores	3–5	84.6
	PSI risk classes	IV- V	46.2
Positive predictive values for mortality, %			
	A-DROP scores	3–5	33.3
	CURB-65 scores	3–5	0
	PSI risk classes	IV- V	50
Negative predictive values for mortality, %			
	A-DROP scores	3–5	61.1
	CURB-65 scores	3–5	57.9
	PSI risk classes	IV- V	85.7

*In PSI, relations of risk classes to net points are as follows:

I: 0, II: 1–70, III: 71–90, IV: 91–130, V: >131.

Table 4 legend - PCP, *Pneumocystis* pneumonia; PSI, pulmonary severity index.

### Comparison of the characteristics between survivors and non survivors

The lactate dehydrogenase (LDH) value tended to be higher and Alb/BUN tended to be lower in the non survivor group compared with the survival group, but this was not statistically significant. There were no significant differences in serum β-d-glucan (β-DG), Krebs von den Lungen 6 (KL-6), body mass index (BMI) between the two groups. However, both the time between the initial visit and establishment of a diagnosis and the time between the initial visit and starting PCP therapy were significant much shorter in the survivor than non survivor group (Table 5).

**Table 5:** Patient characteristics on admission by prognostic outcome

Item	Survivors (n = 13)	Non-survivors (n = 8)	p
Age	69.4 (8.9)	74.9 (9.3)	0.193
BMI (kg/m^2^)	22.3 (3.5)	22.5 (1.7)	0.872
β-DG (pg/ml)	93.8 (161)	82 (64.2)	0.846
KL-6 (U/ml)	771 (461)	1588 (1417)	0.104
LDH (IU/l)	521 (276)	799 (329)	0.051
CRP (mg/dl)	8.22 (4.35)	9.62 (4.92)	0.502
Alb/BUN	0.211 (0.133)	0.119 (0.054)	0.078
Lymphocytes (/μl)	1203 (1011)	752 (389)	0.28
Neutrophils (/μl)	7181 (3012)	7511 (3083)	0.825
WBC (/μl)	9323 (3581)	8700 (3103)	0.7
Interval from admission to PCP diagnosis (days)			
	4 (2.83)	7.88 (3.56)	0.012
Interval from admission to start PCP-specific treatment (days)			
	1.77 (2.74)	6.88 (4.67)	0.005

## Discussion

There are some widely used prognostic guidelines for CAP. These systems appear to be useful in assisting physicians to make more rational decisions regarding the need for admission [13-15]. Patient mortalities in the risk groups 3–5 on A-DROP and CURB-65, and IV-V on PSI have previously been reported as 11.5–23.3%, 11.6–21.0% and 12.5–29.2%, respectively [16-19]. A striking fact is that the majority of the PCP patients were categorized as mild to moderate by these guidelines and resulted in respiratory failure, and poor outcomes. We emphasize that mortality prediction in PCP is not correct when these conventional guidelines for CAP are applied, even when PCP develops in the setting of CAP. Also, these guidelines definitely underestimate the severity of PCP as CAP. The important issue is why these guidelines cannot correctly estimate PCP severity. PCP without HIV infection shows quite different clinical pictures compared to PCP with HIV infection. PCP with HIV occurs slowly and gradually [20]. On the other hand, PCP without HIV is typically more acute and severe than when associated with AIDS [10], often resulting in acute respiratory failure requiring a need for mechanical ventilation. We suppose that this results from the differences of pathologic mechanisms between PCP with and without HIV. It is evident that PCP without HIV is an allergic reaction originating from *Pneumocystis jirovecii. Pneumocystis* elicits many kinds of immune responses, including those by lymphocytes, macrophages, neutrophils, dendritic cells, and epithelial cells [1,21]. There is now a considerable body of evidence showing that immune and inflammatory responses to *Pneumocystis* can have harmful as well as beneficial effects on host lungs.

Another reason why the mortality rate of PCP without HIV remains high is presumably that it is difficult to diagnose PCP according to nonspecific signs, symptoms and/or no reliable culture. Bollée et al. documented that the leading symptoms of PCP in HIV-uninfected cancer patients were fever (85.7%), dyspnea (78.6%), cough (57.1%), and all three symptoms (44.6%) on diagnosis [5], and 14.3% of the patients showed only one symptom. In our study, 4 out of 21 patients (19%) were asymptomatic. In addition, 6/21 patients (28.6%) showed abnormality in chest X-ray on admission. It is possible that steroids and immunosuppressive drugs could mask fever and general fatigue on the initial visit. We strongly believe that clinicians are unable to diagnose non-HIV PCP by clinical picture or chest X-ray alone. The association with *P. jirovecii* cysts has been reported in HIV-uninfected PCP to be one tenth of that in HIV-PCP [4]. Therefore, the sensitivity of conventional staining methods for diagnosis of HIV-uninfected PCP is lower than that for PCP with HIV. Our study demonstrated the sensitivity of conventional staining to be 23.8%. While Diff-Quik staining is highly sensitive, it requires considerable technical expertise [22]. It is likely that physicians are unable to diagnose PCP without HIV soon enough due to the reasons mentioned above.

A clue for making the early diagnosis of PCP is serum β-d-gulcan (β-DG) and chest CT findings. Tasaka et al. reported the β-DG could be a serum indicator for the diagnosis of PCP with the cut-off value of 31 pg/ml [23,24]. In our study, the sensitivity of β-DG in diagnosing PCP was 10/21 (47.6%) setting the cut-off value at 31 pg/ml. We suggest that testing β-DG is effective for diagnosis of PCP. In testing, BAL is also well known to be more sensitive than IS, as many physicians previously reported [23,25]. In terms of a radiological approach, high resolution computed tomography (HRCT) should be performed if PCP is suspected. It is commonly known that chest CT shows ground glass appearance with a panlobular pattern or so-called crazy paving appearance in PCP patients [26,27]. These findings are also found in viral pneumonias, mycoplasmal pneumonia, alveolar hemorrhage, methotrexate pneumonia, and others. However, where the patient’s background and characteristics are conducive, the presence of PCP should be suspected.

Conventional guidelines for CAP have recommended that clinical outcomes should be evaluated three days after initial therapy has been started [13,28-33]. In our study, 12 of the 13 (92.3%) patients who received accurate anti-PCP therapy within 3 days from initial visit were cured. Ten of the 12 (83.3%) patients received empiric therapy for PCP based on patient characteristics, laboratory data and radiological findings on HRCT. On the other hand, 7 of the 8 (87.5%) patients who received anti-PCP therapy that was initiated after day 4 died. PCP without HIV tends to develop acute respiratory failure and results in a more severe, acute form of acute respiratory distress syndrome (ARDS) than PCP with HIV. Thus, only three days of doctor’s delay in starting PCP therapy could be fatal as our study showed.

The limitation of our study is that it is a retrospective analysis in a very small population. Retrospective studies may be less reliable in terms of the data collected, particularly for data such as physical examination. A prospective study should be carried out and with more cases.

## Conclusions

In conclusion, we suggest that conventional prognostic guidelines for CAP might underestimate the severity of HIV-uninfected PCP. Physicians should be aware of the possibility that PCP may occur in non-HIV patients having malignancy or rheumatic disease, receiving steroid and/or immunosuppressive therapy. The most important factor for improving the mortality of PCP without HIV could be the time when anti-PCP therapy is started.

## Competing interests

The authors declare that they have no competing interests.
